# Baseline DISE Anatomy Predicts Jaw-Thrust Responsiveness in Obstructive Sleep Apnea

**DOI:** 10.3390/life16030456

**Published:** 2026-03-11

**Authors:** Wei-Hung Chang, Kuan-Pen Yu, Li-Kuo Kuo, Chung Lee

**Affiliations:** 1 Department of Critical Care Medicine, MacKay Memorial Hospital, Taipei 10449, Taiwan; 2 Department of Medicine, Taipei Tzu Chi Hospital, Buddhist Tzu Chi Medical Foundation, Taipei 10412, Taiwan

**Keywords:** jaw-thrust responsiveness, VOTE scoring, multilevel obstruction, tongue-base collapse, hypopharyngeal collapse, mandibular advancement simulation, DISE phenotyping, non-CPAP therapy selection

## Abstract

Background: Drug-induced sleep endoscopy (DISE) with a jaw-thrust maneuver is used to simulate mandibular advancement in obstructive sleep apnea (OSA), yet determinants of functional airway improvement remain incompletely defined. Objective: To identify clinical, polysomnographic, and baseline DISE anatomic factors associated with jaw-thrust responsiveness. Methods: We conducted a single-center retrospective observational study of adults with polysomnography-confirmed OSA who underwent DISE with paired baseline and jaw-thrust VOTE assessments between 1 January 2015 and 31 December 2025 (n = 355). Jaw-thrust responsiveness was defined a priori as a within-subject reduction in the number of obstructed VOTE sites (grade ≥ 1). Multivariable logistic regression was used to identify independent correlates within a prespecified explanatory modeling framework. The study was approved by the Institutional Review Board of Taipei Tzu Chi Hospital (protocol 14-IRB079), with the need for informed consent being waived. Results: Jaw thrust reduced overall obstruction burden from two (two to three) to one (one to two) sites (Wilcoxon *p* < 0.001). Hypopharyngeal levels demonstrated the greatest improvement, particularly at the tongue base (39.2% to 7.6%) and epiglottis (23.9% to 5.4%) (both *p* < 0.001). Overall, 62.8% met responder criteria and 18.9% achieved complete normalization. In multivariable analysis (n = 272), baseline tongue-base collapse (adjusted odds ratio [aOR] 2.46, 95% CI 1.20–5.04) and greater baseline multilevel obstruction burden (aOR 1.85 per SD, 95% CI 1.19–2.85) were independently associated with responsiveness, whereas conventional PSG severity metrics were not. Conclusions: In adults with OSA, jaw-thrust responsiveness during DISE is more strongly associated with baseline anatomic phenotype than with global PSG severity. Standardized DISE functional assessment may provide complementary information to support phenotype-informed selection of non-CPAP therapies, pending prospective validation.

## 1. Introduction

Obstructive sleep apnea (OSA) is highly prevalent and is associated with recurrent upper-airway narrowing during sleep that can drive intermittent hypoxemia and sleep fragmentation, with downstream neurocognitive symptoms and cardiometabolic risk that have negative impacts at both the individual and population levels [1,2,3]. Recent global estimates suggest that OSA affects nearly one billion adults worldwide, with moderate-to-severe disease present in approximately 425 million individuals, underscoring its substantial public health impact. Moreover, contemporary epidemiological analyses indicate rising prevalence across both developed and developing regions, partly driven by aging populations and increasing obesity rates. In routine care, this translates into sustained clinical and operational burden: large volumes of patients are evaluated with polysomnography (PSG), many require long-term therapy, and treatment selection has direct implications for symptom control, cardiovascular risk mitigation, safety (e.g., vigilance-related outcomes), and downstream resource utilization. Beyond individual-level morbidity, untreated OSA is associated with increased cardiovascular events, metabolic dysfunction, neurocognitive impairment, and accident risk, contributing to substantial healthcare utilization and economic cost. Although the apnea–hypopnea index (AHI) remains central to OSA diagnosis and severity stratification, it does not uniquely determine mechanism or treatment responsiveness, and patients with similar PSG severity can have meaningfully different anatomic contributors, physiologic traits, symptom profiles, and responses to therapy [4,5,6]. This heterogeneity creates a practical problem for workflow: after the PSG report is finalized, clinicians must select among multiple management pathways, yet the information used to guide anatomy-directed decisions is often indirect.

Current practice patterns therefore vary, especially when continuous positive airway pressure (CPAP) is declined, poorly tolerated, or insufficiently effective. PSG provides a physiologic summary of event frequency and sleep-related consequences, but it does not localize the dominant site(s) of collapse or indicate which segments are functionally modifiable for a given patient [4,5,6]. As a result, non-CPAP strategies, including upper-airway surgery, oral appliance therapy, and other anatomy-directed or combination approaches, are frequently chosen under uncertainty, with real-world trade-offs between expected benefit, procedural risk, patient preference, and resource allocation. In this setting, there is a persistent need for clinically usable phenotyping tools that can reduce trial-and-error care and better align mechanism with therapy selection.

Drug-induced sleep endoscopy (DISE) has been adopted as a pragmatic method to bridge this gap by enabling dynamic visualization of upper-airway behavior under sedated, sleep-like conditions [7]. Standardized reporting frameworks, most prominently the VOTE classification (velum, oropharynx, tongue base, epiglottis), have improved communication across centers and support more reproducible description of collapse patterns at defined anatomic levels [8,9]. However, DISE remains protocol-sensitive. Findings may be influenced by sedation depth, scoring conventions, inter-rater variability, and maneuver execution, contributing to ongoing debate regarding standardization and the translation of DISE phenotypes into consistently improved patient outcomes [10,11]. Recent adult DISE guidelines have further formalized indications and procedural considerations (including sedation strategy and maneuver standardization), reinforcing the need to interpret DISE findings within a protocol-governed framework [12]. These limitations do not negate DISE’s clinical value, but they raise an important operational point: if DISE is to function as a reproducible decision-support tool rather than purely descriptive documentation, its functional signals must be systematically characterized and linked to clinically actionable treatment pathways.

Within DISE, the jaw-thrust maneuver is commonly used as a functional test because advancing the mandible can enlarge the retrolingual airway and alter downstream hypopharyngeal behavior, conceptually simulating the mechanism of mandibular advancement therapy [13]. Oral appliance therapy is an established non-CPAP option supported by evidence syntheses and professional society guidance, yet clinical response is variable and depends on patient-specific anatomy and physiology [14,15]. Consistent with this, several studies have suggested that DISE-based mandibular advancement simulation may help identify patients more likely to respond to oral appliance therapy, but reported associations differ across cohorts, scoring approaches, and outcome definitions [13,14,15,16]. Competing interpretations persist. One perspective emphasizes that jaw-thrust response is predominantly an anatomic phenomenon driven by baseline retrolingual or hypopharyngeal obstruction, implying that DISE phenotype should be the primary determinant of responsiveness [13,14]. Another perspective argues that global disease severity and broader physiologic traits may be equally important, potentially limiting the discriminative value of jaw-thrust findings alone for treatment triage [15]. In practice, clinicians often use jaw thrust as a real-time “screen,” yet the determinants of that screen—and the extent to which they can be explained by routinely available clinical and PSG variables versus DISE-defined anatomy—remain incompletely defined.

Accordingly, the key unresolved issue is not whether jaw thrust can change airway appearance during DISE, but which patient and baseline anatomic factors are associated with clinically meaningful improvement during the maneuver, and whether DISE phenotype provides incremental decision-relevant information beyond standard PSG severity metrics in real-world care. Addressing this gap is particularly relevant in a single-center setting, where DISE workflow, scoring conventions, and maneuver execution can be relatively consistent, enabling a clearer assessment of determinants of jaw-thrust responsiveness under a defined operational protocol.

Therefore, we conducted a single-center retrospective observational study of adults with PSG-confirmed OSA who underwent DISE with baseline and jaw-thrust VOTE scoring.

The general objective was to evaluate determinants of jaw-thrust responsiveness during DISE.

The specific objectives were as follows:

(1) To quantify the within-subject change in obstruction burden during jaw thrust;
(2) To identify clinical and polysomnographic predictors of responsiveness;
(3) To determine whether the baseline DISE-defined anatomic phenotype provides incremental information beyond global PSG severity metrics.

We hypothesized that jaw-thrust responsiveness would be more strongly associated with baseline DISE-defined anatomic phenotype, particularly tongue-base involvement and multilevel obstruction burden, than with global PSG-derived severity measures.

Clarifying these determinants may strengthen the role of standardized DISE functional maneuvers as phenotype-informed decision-support tools for individualized non-CPAP treatment selection.

## 2. Materials and Methods

### 2.1. Study Design and Setting

This was a single-center retrospective observational study conducted at Taipei Tzu Chi Hospital, Buddhist Tzu Chi Medical Foundation (Taiwan). The study was embedded in routine clinical care for adult patients evaluated for obstructive sleep apnea (OSA), in which overnight polysomnography (PSG) and drug-induced sleep endoscopy (DISE) were performed as part of the standard diagnostic workflow. The study period spanned 1 January 2015 through 31 December 2025.

The analytic unit was the DISE encounter, with paired baseline and jaw-thrust VOTE assessments recorded within the same session. This study was designed and reported in accordance with the Strengthening the Reporting of Observational Studies in Epidemiology (STROBE) statement [17].

Consecutive eligible encounters during the study period were included, without sampling.

### 2.2. Study Population

The inclusion criteria were as follows:

1. Age ≥ 18 years
2. PSG-confirmed OSA defined as apnea–hypopnea index (AHI) ≥ 5 events per hour, according to American Academy of Sleep Medicine (AASM) criteria [18]
3. DISE performed with baseline VOTE assessment
4. Documented jaw-thrust assessment during the same DISE session

The exclusion criteria included the following:

5. Prior upper-airway surgery potentially altering collapse pattern
6. Prior mandibular advancement therapy
7. Predominant central sleep apnea
8. Incomplete PSG data preventing severity classification
9. Inadequate sedation depth precluding reliable DISE interpretation

Among all available DISE encounters during the study period, 355 met inclusion criteria.

The sample size was determined by available eligible encounters during the defined study period. No a priori power calculation was performed due to the retrospective design.

### 2.3. Data Source and Variables

All variables were obtained from a structured, de-identified institutional clinical dataset derived from routine PSG and DISE assessments.

#### 2.3.1. Primary Outcome

The primary outcome was jaw-thrust responsiveness, defined a priori as a within-subject reduction in the number of obstructed VOTE sites during jaw thrust compared with baseline.

#### 2.3.2. Secondary Outcomes

Secondary outcomes included the following:

- Site-specific complete resolution of collapse
- Site-specific ordinal improvement
- Complete global normalization (0 obstructed sites)

#### 2.3.3. Predictor Variables

Demographic and anthropometric variables included age, gender (replacing sex), body mass index (BMI), and neck circumference. Although components of the STOP-BANG questionnaire were available, the complete STOP-BANG score was not consistently recorded and therefore was not used as an inclusion or stratification variable.

Symptom burden was assessed using the Epworth Sleepiness Scale (ESS) [19,20,21].

PSG-derived variables included AHI, oxygen desaturation index, arousal index, mean oxygen saturation, oxygen saturation nadir, sleep efficiency, sleep-stage composition, and positional parameters. All PSG variables were scored and interpreted according to the American Academy of Sleep Medicine scoring manual [18].

DISE variables included baseline and jaw-thrust VOTE findings. Collapse severity was graded using a standardized three-point scale (0 = no collapse, 1 = partial, 2 = complete). For binary analyses, obstruction was defined as grade ≥ 1.

### 2.4. Procedures and Measurements

DISE was performed under monitored anesthesia care using propofol administered via continuous infusion and titrated to achieve a stable moderate-to-deep sedation state. The target level corresponded to loss of verbal responsiveness with preservation of spontaneous ventilation, approximating a Modified Observer’s Assessment of Alertness/Sedation (MOAA/S) score of 2–3.

Sedation was initiated with incremental boluses followed by continuous infusion, typically within a maintenance range of 50–150 μg/kg/min, adjusted according to respiratory pattern and hemodynamic stability.

Sedation depth was assessed clinically based on responsiveness, respiratory regularity, and the absence of purposeful movement. Bispectral index monitoring was not routinely used during the study period.

Jaw-thrust assessment was performed only after a stable sedation plateau had been achieved for at least 60–90 s without fluctuation in respiratory pattern, in order to minimize transient sedation-related variability.

All procedures were conducted according to a standardized institutional DISE protocol, and no formal modifications to the sedation framework or procedural workflow were introduced during the study period.

DISE scoring reliability

All DISE examinations were performed and interpreted by board-certified otolaryngologists with formal training in the VOTE classification system. Prior to the study period, operators underwent structured calibration sessions using representative video cases to standardize interpretation of collapse severity (grades 0–2) across VOTE levels.

Scoring was conducted in real time during the procedure according to predefined grading criteria. In cases of uncertainty, findings were reviewed immediately with a second experienced operator, and consensus was reached before final documentation.

Although formal inter-rater agreement statistics were not prospectively recorded in this retrospective dataset, institutional scoring conventions and periodic departmental case reviews were maintained throughout the study period to enhance interpretive consistency.

The same core group of operators performed the majority of procedures during the study period.

### 2.5. Missing Data Handling

Missing data were evaluated descriptively. Missingness primarily reflected incomplete historical PSG documentation rather than outcome-related factors. Complete-case analysis was used for multivariable modeling. Although multiple imputation was not performed, sensitivity analyses excluding duplicated identifiers were conducted to assess robustness. Results should be interpreted cautiously if missingness was not completely at random.

Patterns of missingness were examined descriptively. Because missing data were confined to selected PSG parameters and were not associated with responder status in exploratory comparisons, complete-case analysis was considered appropriate for the explanatory modeling framework.

### 2.6. Statistical Analysis

Continuous variables were summarized as the mean ± SD or median (IQR), depending on distribution. Categorical variables were summarized as counts and percentages.

Paired comparisons between baseline and jaw-thrust conditions were conducted using McNemar’s test for binary variables and the Wilcoxon signed-rank test for ordinal variables.

Multivariable logistic regression was used to identify independent predictors of jaw-thrust responsiveness. Candidate predictors were prespecified based on clinical plausibility and prior literature. Continuous predictors were standardized to one standard deviation unit. Categorical variables were coded as binary indicators. Results were reported as adjusted odds ratios (aORs) with 95% confidence intervals.

All candidate variables were entered simultaneously into the multivariable model without stepwise selection in order to preserve theoretical consistency with the prespecified explanatory framework. Collinearity was assessed using variance inflation factors (VIFs), and no clinically significant multicollinearity was identified.

Binary predictors were coded as 1 (presence) and 0 (absence), with reference categories defined a priori.

The multivariable model was specified as an explanatory model to estimate adjusted associations rather than to derive a clinical prediction score. Accordingly, model discrimination metrics (e.g., AUC) were not treated as primary endpoints, and no internal validation procedures were prespecified.

Given the sample size relative to the number of predictors, the events-per-variable ratio was considered adequate for stable estimation.

### 2.7. Data Availability

The data are available from the corresponding author upon reasonable request. Access is subject to institutional review, applicable data-use agreements, and privacy regulations.

### 2.8. Ethics Approval

This study was approved by the Institutional Review Board of Taipei Tzu Chi Hospital, Buddhist Tzu Chi Medical Foundation (protocol code 14-IRB079; approval date 17 November 2025). The need for informed consent was waived due to the retrospective design of the study and the use of de-identified clinical data.

## 3. Results

### 3.1. Study Flow and Analysis Denominators

**Figure 1:**
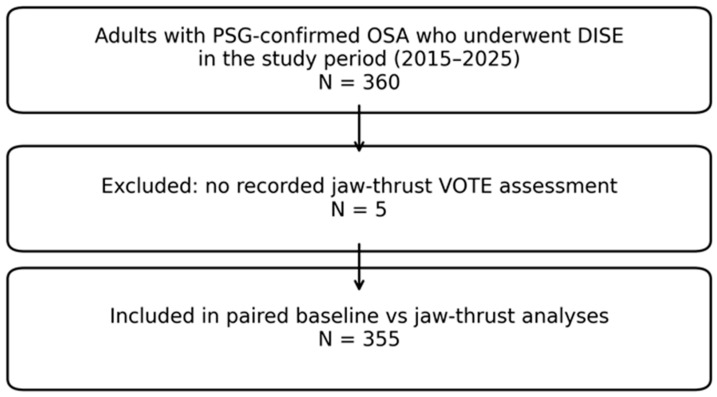
Study flow diagram for the analytic cohort. Abbreviations: DISE, drug-induced sleep endoscopy; OSA, obstructive sleep apnea; PSG, polysomnography; VOTE, velum–oropharynx–tongue base–epiglottis.

During the study period (1 January 2015 to 31 December 2025), 360 adults with PSG-confirmed OSA underwent DISE. Of these, 355/360 (98.6%) had a recorded jaw-thrust VOTE assessment and were included in paired baseline-versus–jaw-thrust analyses (Figure 1). Unless otherwise specified, paired DISE analyses were conducted in 355/355 participants. Selected PSG variables had incomplete availability: sleep efficiency, desaturation index, arousal index, and mean oxygen saturation were available in 354/355 participants, and oxygen saturation nadir was available in 272/355 participants. The multivariable logistic regression model for jaw-thrust responsiveness was therefore estimated in the complete-case subset (272/355 participants).

The final analytic sample for paired DISE comparisons included 355 participants. However, the multivariable logistic regression model was estimated in the complete-case subset (n = 272) due to missing data in selected PSG variables, primarily the oxygen saturation nadir. No additional exclusions were applied beyond variable-specific missingness.

Paired DISE analyses were conducted for n = 355 participants; multivariable models used the complete-case subset (n = 272) due to variable-specific missingness in selected PSG parameters (primarily SpO_2_ nadir).

### 3.2. Baseline Characteristics

Baseline demographic, anthropometric, and PSG characteristics of participants with recorded jaw-thrust assessment are summarized in Table 1 (n = 355). The mean age was 53.2 ± 11.8 years, and 282/355 (79.4%) were male. Median AHI was 30.8 (19.4–50.2) events/h. OSA severity was mild in 54/355 (15.2%), moderate in 118/355 (33.2%), and severe in 183/355 participants (51.5%). The oxygen saturation nadir was available in 272/355 participants and averaged 79.1 ± 8.9%.

### 3.3. Objective 1: Within-Subject Change in Obstruction Burden During Jaw Thrust

**Figure 2:**
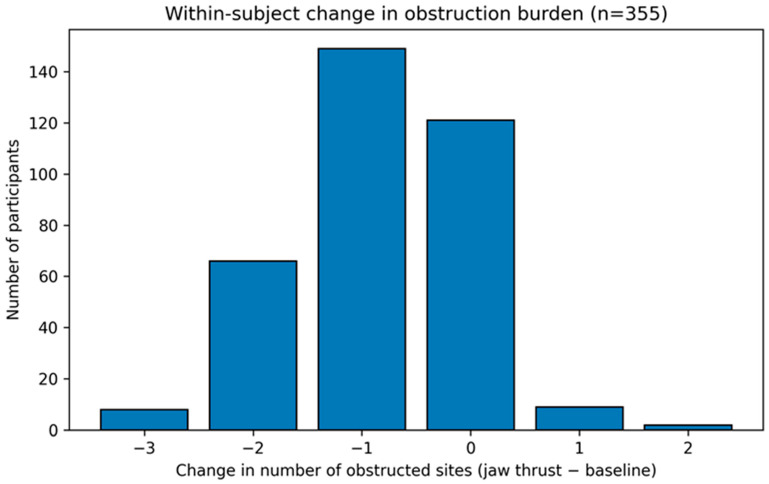
Distribution of within-subject change in obstruction burden (Δ obstructed sites). Δ obstructed sites is defined as (jaw-thrust − baseline) number of obstructed VOTE sites; negative values indicate a reduction in obstruction burden. Denominator: n = 355.

We first quantified the within-subject change in overall obstruction burden during jaw thrust. Changes in paired DISE obstruction burden and responder status are shown in Figure 2 and Table 2. The number of obstructed VOTE sites decreased from two (two to three) at baseline to one (one to two) during jaw thrust (Wilcoxon *p* = 3.67 × 10^−37^). Multilevel obstruction (two or more sites) decreased from 283/355 (79.7%) at baseline to 132/355 (37.2%) during jaw thrust (exact McNemar *p* = 5.98 × 10^−40^). Overall, 223/355 (62.8%) participants met the prespecified definition of jaw-thrust responsiveness (reduced number of obstructed sites), and 67/355 (18.9%) had complete normalization (zero obstructed sites) during jaw thrust.

In the complete-case multivariable model (n = 272, reflecting variable-specific missingness in selected PSG parameters), baseline obstruction burden (adjusted odds ratio [aOR] 1.85 per SD, 95% confidence interval [CI] 1.19–2.85; *p* = 0.006) and baseline tongue-base collapse (aOR 2.46, 95% CI 1.20–5.04; *p* = 0.014) were associated with responder status.

**Figure 3:**
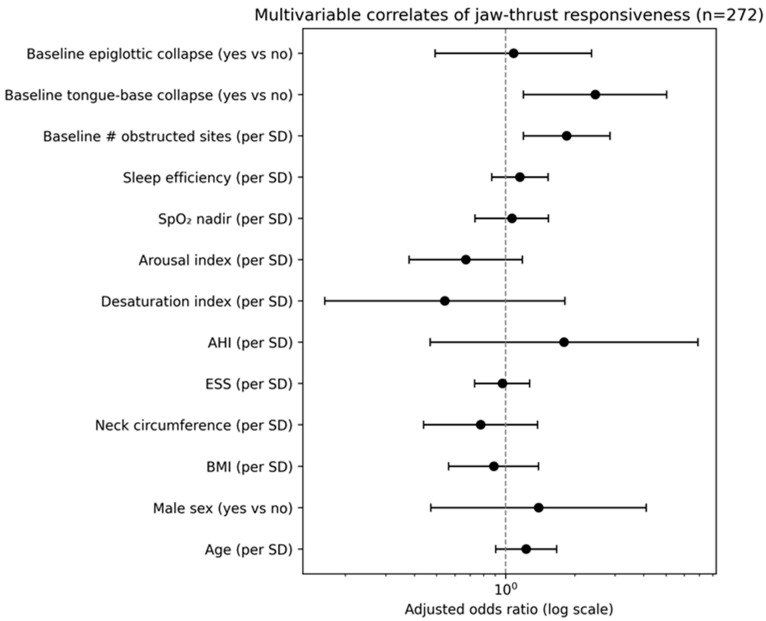
Forest plot of adjusted odds ratios for jaw-thrust responsiveness. Adjusted odds ratios are from the multivariable logistic regression model (n = 272). Continuous predictors are scaled per 1 SD. Abbreviations: aOR, adjusted odds ratio; CI, confidence interval.

The adjusted associations between candidate predictors and jaw-thrust responsiveness are shown in Figure 3 and Table 3.

### 3.4. Objective 1 (Site-Level): Site-Specific Changes in VOTE Severity

We then examined site-specific changes in collapse severity and resolution across the four VOTE levels.

**Figure 4:**
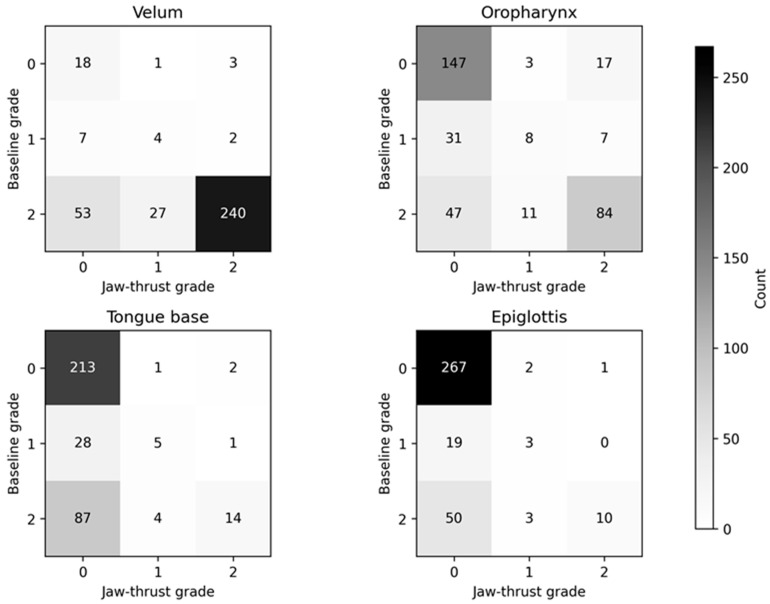
Severity transition matrices (baseline → jaw thrust) by VOTE site (n = 355). Cells show the number of participants transitioning between ordinal collapse grades (0 = no collapse, 1 = partial collapse, 2 = complete collapse). Abbreviations: VOTE, velum–oropharynx–tongue base–epiglottis.

Site-specific changes in DISE collapse severity between baseline and jaw-thrust assessments are summarized in Figure 4 and Table 4 (n = 355). Using a binary definition of obstruction (grade ≥ 1), the prevalence of collapse decreased at the velum from 333/355 (93.8%) to 277/355 (78.0%), at the oropharynx from 188/355 (53.0%) to 130/355 (36.6%), at the tongue base from 139/355 (39.2%) to 27/355 (7.6%), and at the epiglottis from 85/355 (23.9%) to 19/355 (5.4%) (exact McNemar *p* values for each site are shown in Table 4).

Binary paired comparisons were assessed using exact McNemar tests; ordinal severity transitions were assessed using Wilcoxon signed-rank tests.

**Figure 5:**
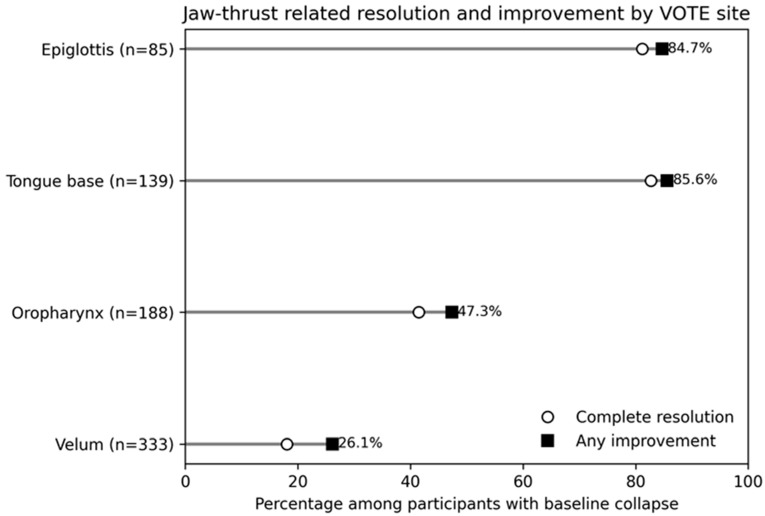
Jaw-thrust-related resolution and improvement rates by VOTE site. Percentages are calculated among participants with baseline collapse at the corresponding site (grade ≥ 1). Markers indicate complete resolution and any improvement; labels denote any-improvement percentages. Abbreviations: VOTE, velum–oropharynx–tongue base–epiglottis.

Among participants with baseline collapse at each VOTE site, complete resolution and any improvement during jaw thrust are shown in Figure 5 and Table 5. Complete resolution occurred in 60/333 (18.0%) at the velum, 78/188 (41.5%) at the oropharynx, 115/139 (82.7%) at the tongue base, and 69/85 (81.2%) at the epiglottis. Improvement occurred in 87/333 (26.1%), 89/188 (47.3%), 119/139 (85.6%), and 72/85 participants (84.7%), respectively.

In the prespecified sensitivity analysis excluding encounters with duplicated de-identified identifiers, 349 participants remained eligible. In this subset, 222/349 (63.6%) met responder criteria and 67/349 (19.2%) had complete normalization during jaw thrust. The complete-case multivariable model in the sensitivity subset included 268 participants; baseline obstruction burden (aOR 1.89 per SD, 95% CI 1.22–2.94; *p* = 0.005) and baseline tongue-base collapse (aOR 2.36, 95% CI 1.14–4.89; *p* = 0.021) remained associated with responder status.

### 3.5. Objectives 2–3: Predictors of Jaw-Thrust Responsiveness

To identify independent predictors of jaw-thrust responsiveness and evaluate the incremental contribution of baseline DISE-defined anatomy beyond PSG-derived severity metrics, multivariable logistic regression models were constructed.

The model was estimated in the complete-case subset (n = 272), reflecting variable-specific missingness in selected PSG parameters, primarily the oxygen saturation nadir. No additional clinical exclusions were applied beyond missing covariate data.

In the adjusted model, greater baseline obstruction burden (aOR 1.85 per SD, 95% CI 1.19–2.85; *p* = 0.006) and baseline tongue-base collapse (aOR 2.46, 95% CI 1.20–5.04; *p* = 0.014) were independently associated with responder status. In contrast, conventional PSG severity metrics, including AHI and oxygen desaturation index, were not independently associated with jaw-thrust responsiveness after adjustment.

These findings support the hypothesis that jaw-thrust responsiveness during DISE is more strongly associated with baseline anatomic phenotype—particularly tongue-base involvement and multilevel obstruction burden—than with global PSG severity indices alone.

Although VOTE collapse patterns (anteroposterior, lateral, concentric) were recorded in some encounters, pattern labels were not consistently available across the full study period; therefore, primary analyses focused on severity grades (0–2) and obstruction burden.

### 3.6. Sensitivity Analyses

Prespecified sensitivity analyses were conducted to evaluate the robustness of the primary findings.

First, to address the potential impact of duplicated de-identified identifiers in the dataset, analyses were repeated after excluding encounters with repeated identifiers. In this sensitivity subset, 349 participants remained eligible for paired DISE analyses. Among these, 222/349 (63.6%) met the prespecified responder definition, and 67/349 (19.2%) achieved complete normalization during jaw thrust.

The complete-case multivariable logistic regression model in this sensitivity subset included 268 participants. In this model, greater baseline obstruction burden (aOR 1.89 per SD, 95% CI 1.22–2.94; *p* = 0.005) and baseline tongue-base collapse (aOR 2.36, 95% CI 1.14–4.89; *p* = 0.021) remained independently associated with responder status. The direction and magnitude of associations were consistent with those observed in the primary analysis.

These findings indicate that the observed associations between the baseline DISE-defined anatomic phenotype and jaw-thrust responsiveness were stable across analytic subsets and were not materially influenced by duplicated identifiers or variable-specific missingness. Overall, sensitivity analyses yielded materially similar estimates, supporting robustness of the primary conclusions.

Additional sensitivity analyses were performed using stricter responder definitions, including (i) a two-or-more-site reduction in obstruction burden and (ii) complete normalization (0 obstructed sites). The direction and relative magnitude of associations between baseline tongue-base collapse, multilevel obstruction burden, and responsiveness remained consistent across these alternative definitions, although effect sizes were attenuated due to reduced event counts.

## 4. Discussion

This study aimed to (i) quantify within-subject changes in VOTE obstruction during jaw thrust, (ii) identify clinical/PSG predictors of responsiveness, and (iii) determine whether baseline DISE anatomy provides incremental information beyond PSG severity.

In this single-center retrospective cohort of adults with PSG-confirmed OSA undergoing DISE, the jaw-thrust maneuver was associated with a within-subject reduction in overall obstruction burden, with the most prominent improvements observed at hypopharyngeal levels. The marked reduction at the tongue base and epiglottis levels provides mechanistic support for the concept that mandibular advancement primarily modifies the retrolingual and hypopharyngeal airway. Jaw-thrust responsiveness was more strongly associated with baseline DISE-defined anatomy—particularly tongue-base involvement and greater baseline multilevel obstruction burden—than with PSG-derived severity metrics. These findings support the use of standardized DISE functional assessment as a complementary phenotype signal for non-CPAP treatment triage in real-world practice. Notably, improvement at the velum level was comparatively limited, underscoring that jaw thrust does not represent a universal airway rescue maneuver but rather a phenotype-specific functional probe.

A central clinical motivation for assessing jaw-thrust response during DISE is to approximate the functional effect of mandibular advancement and inform selection for oral appliance therapy. Our prespecified definition of a responder—displaying a reduction in the number of obstructed VOTE sites—can be interpreted as a within-subject functional modifiability index, reflecting whether airway anatomy is dynamically responsive to mandibular advancement rather than merely reflecting cross-sectional PSG severity. Prior work suggests that DISE-informed phenotyping and mandibular advancement simulation can support candidate selection for oral appliance therapy [20,21,22,23]. More recent outcome-oriented studies further indicate that DISE-guided selection may improve treatment effectiveness with mandibular advancement devices, although definitions and response thresholds vary across cohorts [22,23]. Our observation that baseline anatomic phenotype correlated more strongly with jaw-thrust responsiveness than PSG severity aligns with emerging evidence that DISE-derived phenotypes may provide incremental predictive value beyond conventional PSG severity indices [24]. At the same time, PSG-based physiology remains clinically relevant; frameworks that derive endotypes or pathophysiologic traits from PSG emphasize that event frequency alone incompletely captures mechanism and treatment responsiveness. Accordingly, the comparatively weaker independent association between conventional PSG severity metrics and jaw-thrust responsiveness in fully adjusted models is consistent with broader views of OSA heterogeneity, in which single PSG summary measures may have limited predictive utility for the specific question of anatomic modifiability [25]. This attenuation may reflect construct mismatch: PSG metrics summarize event frequency and physiological consequences during natural sleep, whereas jaw-thrust responsiveness represents anatomic modifiability under simulated mandibular advancement. These constructs address distinct clinical questions. Contemporary precision-medicine frameworks emphasize that PSG-derived severity indices and physiological endotypes capture partially distinct constructs from anatomical modifiability assessed by dynamic upper-airway evaluation, which may explain the limited independent association between conventional PSG severity metrics and jaw-thrust responsiveness in adjusted models [26,27]. In addition, substantial shared variance exists among PSG-derived parameters (e.g., AHI, desaturation index, arousal index). When baseline DISE-defined multilevel obstruction burden is included in the model, it may absorb phenotype-related variance, thereby diminishing the apparent independent contribution of PSG severity indices. However, dichotomizing responsiveness based solely on reduction in obstructed site count inevitably sacrifices gradational severity information. A transition from complete (grade 2) to partial (grade 1) collapse may represent clinically meaningful improvement without altering site count. Future work may consider weighted obstruction scores or continuous Δsum (VOTE grade) metrics to preserve severity information.

Multiple groups have attempted to formalize DISE-informed selection for non-CPAP therapies using prediction models or phenotypic labeling. Prior efforts to develop DISE-based selection tools for oral appliance treatment and positional therapy highlight that model performance depends on how “response” is defined and measured [28]. Prospective evidence further suggests that DISE-based phenotypic labeling can improve patient selection for mandibular advancement device outcomes, providing a clinically relevant benchmark for interpreting our findings, even though our endpoint reflects functional response during DISE rather than longitudinal treatment efficacy [29]. Earlier clinical series also described DISE as a pragmatic selection tool for mandibular advancement therapy, supporting the concept that maneuver-based assessment may aid candidate triage [30]. In addition, comparisons between manual jaw thrust and temporary mandibular advancement approaches during DISE indicate that the method of mandibular advancement simulation can influence the observed response signal, which is relevant when comparing findings across centers with different protocols [31].

Our observation that upstream collapse can persist despite improvement at downstream levels is compatible with emerging work on collapse coupling at the velopharyngeal level, where interactions between segments may limit the degree of palatal improvement achievable through mandibular advancement alone in some patients [32]. This is also coherent with the historical development of sleep endoscopic assessment, in which procedure technique and scoring conventions have evolved over time, contributing to heterogeneity in reported collapse patterns and maneuver effects across studies [33].

Mechanistically, mandibular advancement is understood to enlarge the retrolingual airway and modify upper-airway soft tissue configuration, providing a physiologic rationale for the pronounced hypopharyngeal improvements observed during jaw thrust. The oral appliance literature emphasizes variable treatment response and the importance of patient selection, reinforcing why a DISE-derived anatomic “treatability” signal is clinically attractive [34,35]. From a diagnostic workflow perspective, the incremental value of DISE relative to awake examination has been debated; evidence comparing awake assessment with DISE for surgical decision making indicates that dynamic evaluation can change treatment concepts, supporting the broader role of DISE in anatomy-directed pathways [36]. Earlier work using sleep nasendoscopy to predict success with mandibular advancement splints similarly supports the concept that endoscopic assessment under sleep-like conditions may inform oral appliance selection [37].

Jaw-thrust responsiveness during DISE should be regarded as a physiological surrogate rather than a guarantee of oral appliance treatment success. Studies examining DISE in patients with incomplete oral appliance response suggest that residual obstruction patterns may remain clinically relevant and may motivate reassessment or combination approaches [38]. Systematic reviews of predictors of oral appliance outcomes further emphasize that prediction remains imperfect and dependent on cohort definitions and outcome ascertainment, supporting cautious interpretation of jaw-thrust responsiveness as one component of an integrated selection strategy rather than a standalone determinant [39]. Procedural conditions may also influence the maneuver signal itself; evidence that modified jaw-thrust techniques can affect sedation depth during DISE is relevant because changes in sedation level could alter airway behavior and potentially confound interpretation if depth is not tightly controlled [40]. Its clinical value lies in providing operational guidance for phenotype-informed triage, not in serving as a standalone predictor of long-term therapeutic efficacy. Sensitivity analyses using stricter responder definitions—such as requiring a two-or-more-site reduction or mandatory tongue-base improvement—may further clarify the robustness of identified predictors.

From an implementation standpoint, our findings support incorporating DISE jaw-thrust assessment as a structured decision-support element when considering mandibular advancement pathways, particularly to identify patients in whom hypopharyngeal collapse is functionally modifiable during mandibular advancement simulation. This is consistent with prospective data suggesting that DISE-driven phenotypic labeling can improve selection for mandibular advancement device outcomes and provides a plausible downstream pathway for clinical utility if maneuver responsiveness correlates with real-world treatment response [41]. However, further standardization of the maneuver approach and response thresholds remains important. Studies using simulation bites during DISE to predict oral appliance outcome illustrate an alternative, potentially more standardized method of mandibular advancement simulation that may reduce operator-dependent variability [42]. Remotely controlled mandibular protrusion during natural sleep has also been proposed as a predictive approach; together, these lines of work motivate comparative studies evaluating which simulation method offers the best balance of feasibility, reproducibility, and predictive validity for routine workflows [43].

Several limitations warrant consideration. First, this study was retrospective and did not include longitudinal outcomes after mandibular advancement therapy; therefore, our results describe functional response during DISE rather than treatment efficacy. Second, jaw-thrust execution and sedation conditions may vary in practice and could influence observed responsiveness; this is particularly relevant given that different screening approaches (manual jaw thrust versus temporary devices) may yield non-identical response signals. Third, selected PSG variables had incomplete availability, and complete-case modeling may introduce selection bias if missingness is not random. Because multivariable analyses were restricted to the complete-case subset (n = 272), and oxygen saturation nadir had the highest proportion of missingness, non-random missingness could have biased PSG parameter estimates toward the null, potentially attenuating their statistical significance. Finally, as a single-center study, external validity may be limited by local case-mix and procedural workflow. Although sedation was protocolized, the absence of objective depth monitoring (e.g., BIS) may introduce variability in airway behavior across individuals [44].

Future studies should prospectively link jaw-thrust responsiveness during DISE to objective mandibular advancement therapy outcomes using prespecified success criteria and standardized advancement magnitude, while also measuring sedation depth to reduce protocol-induced variability. Comparative studies should evaluate whether jaw thrust, simulation bite, or other mandibular advancement simulations provide superior predictive validity and operational feasibility. A protocolized approach, such as titratable mandibular advancement maneuvers during sleep endoscopy, provides a potential framework for standardization and prospective validation across centers [44].

## 5. Conclusions

In this single-center retrospective observational study of adults with PSG-confirmed obstructive sleep apnea, we addressed three prespecified objectives. First, we quantified the within-subject change in obstruction burden during a jaw-thrust maneuver and demonstrated a consistent reduction in overall VOTE-defined obstruction, particularly at hypopharyngeal levels. Second, we identified independent predictors of jaw-thrust responsiveness, showing that greater baseline multilevel obstruction burden and tongue-base collapse were associated with a higher likelihood of functional improvement. Third, we found that baseline DISE-defined anatomic phenotype provided information beyond conventional PSG severity metrics, which were not independently associated with responsiveness in fully adjusted models.

These findings indicate that jaw-thrust responsiveness during DISE is more closely related to baseline anatomic configuration than to global PSG-derived severity indices. As an observational study, our results describe associations rather than causal effects. Clinically, standardized DISE-based functional assessment may serve as a complementary phenotype signal to inform non-CPAP treatment triage, particularly in the context of mandibular advancement strategies; however, prospective validation against longitudinal therapeutic outcomes remains necessary before routine implementation as a predictive tool.

## Figures and Tables

**Table 1:** Baseline characteristics of participants with recorded jaw-thrust DISE assessment (n = 355).

Characteristic	Value
Participants with recorded jaw-thrust DISE assessment, n	355
Age, years	53.2 ± 11.8
Male sex, n (%)	282 (79.4%)
Body mass index, kg/m^2^	26.9 ± 3.7
Neck circumference, cm	38.0 ± 3.2
Epworth Sleepiness Scale	9.3 ± 4.3
Sleep efficiency, %	76.8 ± 12.9 (n = 354)
AHI, events/h	30.8 (19.4–50.2)
Supine AHI, events/h	45.2 (25.6–64.0)
Lateral AHI, events/h	4.3 (0.0–13.6)
Desaturation index, events/h	29.7 ± 22.0 (n = 354)
Arousal index, events/h	29.8 ± 19.9 (n = 354)
Mean SpO_2_, %	93.9 ± 2.3 (n = 354)
SpO_2_ nadir, %	79.1 ± 8.9 (n = 272)
OSA severity (mild), n (%)	54 (15.2%)
OSA severity (moderate), n (%)	118 (33.2%)
OSA severity (severe), n (%)	183 (51.5%)
Diabetes mellitus, n (%)	43 (12.1%)
Hypertension, n (%)	125 (35.2%)
Chronic obstructive airway disease, n (%)	72 (20.3%)
Coronary artery disease, n (%)	22 (6.2%)
Cerebrovascular disease, n (%)	4 (1.1%)

Values are presented as mean ± SD, median (interquartile range), or n (%). Variable-specific denominators are shown where data are incomplete.

**Table 2:** Changes in obstruction burden and multilevel collapse during jaw-thrust assessment (n = 355).

Outcome	Baseline DISE	Jaw Thrust DISE	*p* Value
Number of obstructed VOTE sites (0)	2 (0.6%)	67 (18.9%)	
Number of obstructed VOTE sites (1)	70 (19.7%)	156 (43.9%)	
Number of obstructed VOTE sites (2)	192 (54.1%)	106 (29.9%)	
Number of obstructed VOTE sites (3)	73 (20.6%)	19 (5.4%)	
Number of obstructed VOTE sites (4)	18 (5.1%)	7 (2.0%)	
Multilevel obstruction (≥2 sites)	283 (79.7%)	132 (37.2%)	5.98 × 10^−40^
Responder (reduced number of sites)		223 (62.8%)	
Complete normalization (0 sites)		67 (18.9%)	
Paired comparison for number of sites (Wilcoxon *p*)			3.67 × 10^−37^

Values are n (%) or median (IQR), as appropriate. Abbreviations: DISE, drug-induced sleep endoscopy; VOTE, velum–oropharynx–tongue base–epiglottis. *p* values: multilevel obstruction (≥2 sites), exact McNemar test; number of obstructed sites, Wilcoxon signed-rank test.

**Table 3:** Multivariable logistic regression for jaw-thrust responsiveness (responder vs. non-responder).

Predictor	aOR (95% CI)	*p* Value
Age (per SD)	1.23 (0.91–1.67)	0.185
Male sex (yes vs. no)	1.39 (0.47–4.11)	0.550
BMI (per SD)	0.89 (0.56–1.39)	0.603
Neck circumference (per SD)	0.78 (0.44–1.38)	0.390
ESS (per SD)	0.97 (0.73–1.27)	0.808
AHI (per SD)	1.80 (0.47–6.89)	0.393
Desaturation index (per SD)	0.54 (0.16–1.81)	0.321
Arousal index (per SD)	0.67 (0.38–1.18)	0.167
SpO_2_ nadir (per SD)	1.06 (0.74–1.54)	0.741
Sleep efficiency (per SD)	1.15 (0.87–1.53)	0.317
Baseline number of obstructed sites (per SD)	1.85 (1.19–2.85)	0.006
Baseline tongue-base collapse (yes vs. no)	2.46 (1.20–5.04)	0.014
Baseline epiglottic collapse (yes vs. no)	1.08 (0.49–2.37)	0.845

Abbreviations: aOR, adjusted odds ratio; CI, confidence interval; SD, standard deviation. Odds ratios were derived from multivariable logistic regression; *p* values are from Wald tests. Continuous predictors were standardized per 1 SD. Model estimated in the complete-case subset (n = 272).

**Table 4:** DISE collapse severity by VOTE site during baseline and jaw-thrust assessments (n = 355).

VOTE Site	Baseline: None (0)	Baseline: Partial (1)	Baseline: Complete (2)	Jaw Thrust: None (0)	Jaw Thrust: Partial (1)	Jaw Thrust: Complete (2)	Exact McNemar *p* (≥1)	Wilcoxon *p* (0–2)
Velum	22 (6.2%)	13 (3.7%)	320 (90.1%)	78 (22.0%)	32 (9.0%)	245 (69.0%)	7.36 × 10^−14^	1.68 × 10^−14^
Oropharynx	167 (47.0%)	46 (13.0%)	142 (40.0%)	225 (63.4%)	22 (6.2%)	108 (30.4%)	2.87 × 10^−9^	1.47 × 10^−6^
Tongue base	216 (60.8%)	34 (9.6%)	105 (29.6%)	328 (92.4%)	10 (2.8%)	17 (4.8%)	1.65 × 10^−30^	6.29 × 10^−22^
Epiglottis	270 (76.1%)	22 (6.2%)	63 (17.7%)	336 (94.6%)	8 (2.3%)	11 (3.1%)	2.64 × 10^−17^	9.14 × 10^−14^

Values are n (%). Abbreviations: DISE, drug-induced sleep endoscopy; VOTE, velum–oropharynx–tongue base–epiglottis. *p* values: paired binary obstruction (grade ≥ 1), exact McNemar test; ordinal severity grades (0–2), Wilcoxon signed-rank test.

**Table 5:** Jaw-thrust-related resolution and improvement among participants with baseline collapse at each VOTE site.

VOTE Site	Baseline Collapse (n)	Complete Resolution Under Jaw Thrust, n (%)	Any Improvement Under Jaw Thrust, n (%)
Velum	333	60 (18.0%)	87 (26.1%)
Oropharynx	188	78 (41.5%)	89 (47.3%)
Tongue base	139	115 (82.7%)	119 (85.6%)
Epiglottis	85	69 (81.2%)	72 (84.7%)

Baseline collapse is defined as grade ≥ 1 at baseline DISE. Abbreviations: DISE, drug-induced sleep endoscopy; VOTE, velum–oropharynx–tongue base–epiglottis.

## Data Availability

The data presented in this study are available on reasonable request from the corresponding author. The data are not publicly available due to privacy and ethical restrictions.

## Abbreviations

AHI	apnea–hypopnea index;
AI	arousal index;
BMI	body mass index;
CPAP	continuous positive airway pressure;
DI	desaturation index;
DISE	drug-induced sleep endoscopy;
ESS	Epworth Sleepiness Scale;
ICH-GCP	International Council for Harmonisation—Good Clinical Practice;
IRB	Institutional Review Board;
OSA	obstructive sleep apnea;
PSG	polysomnography;
SpO_2_	peripheral oxygen saturation;
VOTE	velum–oropharynx–tongue base–epiglottis.
